# Mechanical Properties of the Modified Denture Base Materials and Polymerization Methods: A Systematic Review

**DOI:** 10.3390/ijms23105737

**Published:** 2022-05-20

**Authors:** Aftab Ahmed Khan, Muhammad Amber Fareed, Abdulkarim Hussain Alshehri, Alhanoof Aldegheishem, Rasha Alharthi, Selma A. Saadaldin, Muhammad Sohail Zafar

**Affiliations:** 1 Dental Biomaterials Research Chair, College of Applied Medical Sciences, King Saud University, Riyadh 11451, Saudi Arabia; 2 Department of Restorative Dentistry, College of Dentistry, Gulf Medical University, Ajman P.O. Box 4184, United Arab Emirates; prof.mafareed@gmu.ac.ae; 3 Department of Prosthetic Dental Sciences, College of Dentistry, Jazan University, Jazan 45142, Saudi Arabia; dr.alshehri11@gmail.com; 4 Department of Clinical Dental Science, College of Dentistry, Princess Nourah bint Abdulrahman University, P.O. Box 84428, Riyadh 11671, Saudi Arabia; asaldegheishem@pnu.edu.sa (A.A.); rsalharthi@pnu.edu.sa (R.A.); 5 Prosthodontics Division, Schulich School of Medicine and Dentistry, Western University, London, ON N6A 5B9, Canada; ssaadal@uwo.ca; 6 Department of Restorative Dentistry, College of Dentistry, Taibah University, Al Madinah Al Munawarah, Medina 42353, Saudi Arabia; mzafar@taibahu.edu.sa; 7 Department of Dental Materials, Islamic International Dental College, Riphah International University, Islamabad 46000, Pakistan

**Keywords:** denture base, chemical modification, mechanical properties, polymerization, systematic review

## Abstract

Amidst growing technological advancements, newer denture base materials and polymerization methods have been introduced. During fabrication, certain mechanical properties are vital for the clinical longevity of the denture base. This systematic review aimed to explore the effect of newer denture base materials and/or polymerization methods on the mechanical properties of the denture base. An electronic database search of English peer-reviewed published papers was conducted using related keywords from 1 January 2011, up until 31 December 2021. This systematic review was based on guidelines proposed by the Preferred Reporting Items for Systematic Reviews and Meta-Analyses (PRISMA). The search identified 579 papers. However, the inclusion criteria recognized 22 papers for eligibility. The risk of bias was moderate in all studies except in two where it was observed as low. Heat cure polymethyl methacrylate (PMMA) and compression moulding using a water bath is still a widely used base material and polymerization technique, respectively. However, chemically modified PMMA using monomers, oligomers, copolymers and cross-linking agents may have a promising result. Although chemically modified PMMA resin might enhance the mechanical properties of denture base material, no clear inferences can be drawn about the superiority of any polymerization method other than the conventional compression moulding technique.

## 1. Introduction

To date, polymethyl methacrylate (PMMA) is extensively used as denture base material. This unique material is regarded as the material of choice for denture base fabrication due to its low cost and water solubility/sorption, dimensional stability, and ample strength. However, some disadvantages associated with this material are dimensional instability, residual monomer content, and poor mechanical properties, particularly transverse and impact strength. Poor mechanical properties usually cause a fracture in the denture base, insitu and exsitu [1].

Different approaches have been adapted to enhance the mechanical properties of PMMA denture base material. In the past two decades, experiments were conducted with the incorporation of filler particles and fibres of different shapes, sizes, forms, and orientations [2,3]. Subsequently, the use of nanoparticles was accelerated to enhance the weak mechanical properties of denture base polymer [4]. However, nanoparticles tend to agglomerate in a denture polymer matrix. Agglomeration may enhance the hardness of the resin polymer [5]. In contrast, toughness, flexural strength, tensile strength, and other important mechanical properties are largely affected due to the inhomogeneous dispersion of nanoparticles in the matrix system. Moreover, the shape, form, orientation, surface treatment and the interfacial adhesion of nanoparticles with polymer matrix are some of the crucial aspects for consideration [6].

The necessity to produce a resilient, tough, durable and fracture-resistant denture base diverted the attention of the investigators toward chemical modification of the PMMA, experimenting with a novel denture base material or altering the polymerization methods [7]. In recent years, chemical modification has been executed to form a new material with tunable properties. The blending of copolymers with different properties and in different volumetric ratios may strengthen the mechanical properties of denture base PMMA polymer [8,9].

Due to advancements in science and technology, the possibility of enhancing the mechanical properties can be envisaged [10]. The availability of pre-polymerized acrylic resin blocks that can be milled without polymerization shrinkage and 3-D printed denture resin without technical error might help in this aspect [11]. Besides, the use of newer thermoplastic resins such as polycarbonate, polyamide, and Polyetheretherketone (PEEK) with high toughness can also be seen as promising materials [12]. High-performance polymer (BioHPP) based on polyether ketone can offer favourable features for the fabrication of PMMA denture base also [13].

**Figure 1:**
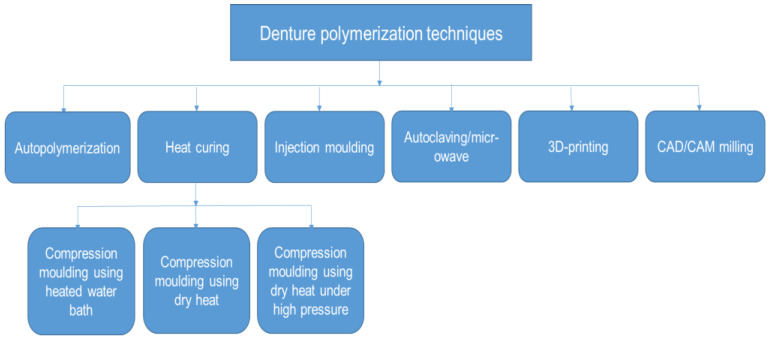
Schematic diagram of the contemporary denture polymerization techniques.

In the traditional polymerization method, polymerization and polymeric chains continue to grow with increasing heat until monomers transform into the polymer [14]. However, a reduction in temperature may decelerate the formation of polymers. Subsequently, residual monomers remain in the polymerized resin [15]. The plasticizing action of the residual monomers can negatively affect the physical, mechanical and biological properties of the denture base [16]. Over the years, curing procedures have been modified to improve the physical and mechanical properties of denture base materials (Figure 1). Recently, the use of processing techniques like injection moulding, microwave energy, autoclaving, heat polymerization under high pressure, CAD-CAM milling and 3-D printing have been proposed. However, little is known about the efficacy of these methods. 

With so many available cross-linking agents, monomers, copolymers and the advent of new denture materials and polymerization methods, a timely systematic review is required to determine the laboratory results of the mechanical properties. Additionally, development in this research area may help the researchers’ effort. Hence, this systematic review aimed to judge, equate, and examine the effects of cross-linking agents, monomers, copolymers and novel polymerization methods used for optimizing denture base material on the mechanical properties.

## 2. Methods

### 2.1. Core Questions

The focused questions of this research were: (1) “Do chemically modified or newer denture base materials have enhanced mechanical properties?” (2) “Are newer denture base polymerization methods better than the traditional ones?’’

### 2.2. Search Strategy

The Medline/PubMed, Web of Science and Scopus databases were last searched on 31 December 2021. Only dental or materials science-related journals were scrutinized electronically and the data were collected for further overview. The keywords used for the search approach are declared in Table 1.

### 2.3. Eligibility Criteria

The published studies with ample sample size and statistically analyzed data were included. The published studies must be laboratory studies with purely mechanical outcomes.

### 2.4. Inclusion and Exclusion Criteria

In vitro studies that aimed to evaluate the mechanical properties, i.e., flexural strength (FS), flexural modulus (FM), impact strength (IS), tensile strength (TS), compressive strength (CS), surface hardness (SH), and fracture toughness (FT) of a novel denture base or chemically modified PMMA denture base material using conventional or novel polymerization methods were included. 

Excluded were in vivo, clinical trials, filler/fibre reinforced denture base, denture repair, and fixed prostheses or overdentures related studies. The review articles, meta-analysis, case report/series, literature reviews, incomplete studies, and articles published in a language other than English were also excluded.

### 2.5. Risk of Bias

The two reviewers independently and critically weighed the methodological quality of each included research [17]. The variables used for quality assessment were: sample fabrication technique, sample size, sample randomization, sample power calculation, blinding of the operator, ISO/ADA standards, and outcome reported (Table 2). If the criteria written in the study were clear, it received a score of “0”. If the required data were vague or uncertain, the score was set as “1”, and if a specific approach was undisclosed, the score was established as “2”. Any divergences in scoring were amicably resolved by consensus between the examiners. If necessary, a third examiner (PV) was consulted in case of disagreement. Papers that scored count 0 to 4, were considered as “low risk of bias”; counts between 5 to 9, as moderate; and between 10 to 14 as high-risk.

## 3. Results

### 3.1. Data Selection

**Figure 2:**
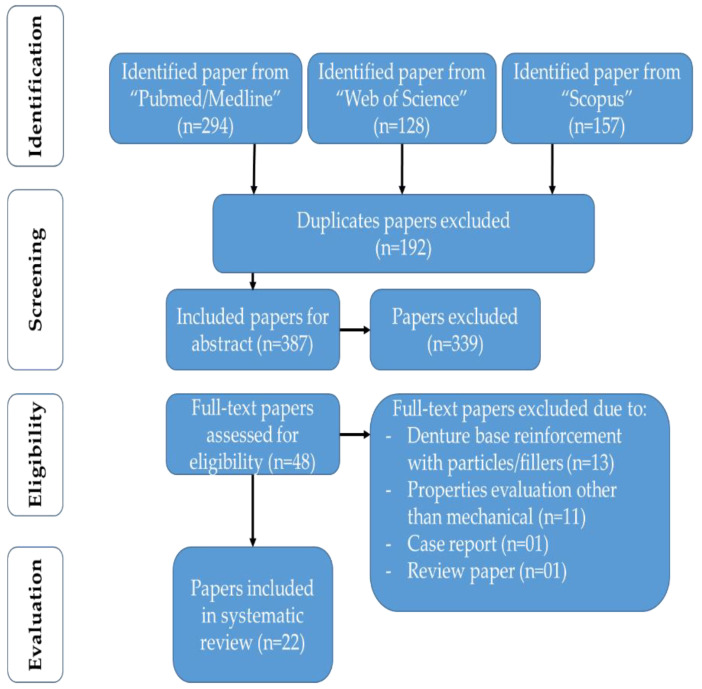
PRISMA flow chart of identified papers and their screening for inclusion in a systematic review.

A total of 579 relevant published papers were retrieved from the electronic data source using search engines. The date range used was 1 January 2011, to 31 December 2021. The obtained results were imported into Endnote X9 software (Thompson Reuters, Philadelphia, PA, USA) and filtered for duplications (192 articles). Subsequently, 387 articles were included for review of their abstracts. Careful abstract reading by five independent reviewers (A.A.K., M.A.F., A.A., R.A. and M.S.Z.) found 99 papers related to filler/fibre reinforcement of denture base material, hence excluded. While 75 papers related to biological or clinical domain were barred from inclusion; 67 papers were excluded due to denture repair/relining/denture teeth/overdenture; 57 papers were excluded due to implant/finite element analysis/fixed prosthesis; 21 papers were removed due to review and case report/series, and 20 papers were disqualified due to denture work/denture cleaners. The remaining 48 titles were thoroughly judged by three pairs of independent reviewers (A.A.K. and M.A.F.; A.H.A. and S.A.S.; A.A. and R.A.). Further, 26 papers were axed due to evaluation of denture base resin properties other than the mechanical, case report, review paper and filler/fibre reinforcement effect. Finally, 22 papers were selected and included that fulfilled the criteria according to the preferred reporting items for systematic reviews and meta-analyses (PRISMA) Statement (Figure 2) [18].

### 3.2. Quality Assessment

From Table 2, it can be agreed that most of the included papers demonstrated a moderate risk of bias (i.e., 20). The remaining two papers demonstrated a low risk of bias. None of the included research showed a high risk of bias. However, the frequent variables missing from the papers were “sample randomization” and “blinding of the operator”. The included papers explicitly mentioned sample fabrication technique, sample size and outcome of the research.

**Table 2:** Characteristics of included studies based on modified CONSORT criteria.

Reference	Sample Fabrication Technique	Sample Size	Sample Randomization	Sample Power Calculation	Testing Standards	Blinding of Operator	Research Finding	Risk of Bias
[19]	0	0	2	2	0	2	0	Moderate
[20]	0	0	2	2	0	2	0	Moderate
[21]	0	0	2	2	0	2	0	Moderate
[22]	0	0	2	2	2	2	0	Moderate
[23]	0	0	2	1	0	2	0	Moderate
[24]	0	0	2	2	2	2	0	Moderate
[25]	0	0	2	2	0	2	0	Moderate
[26]	0	0	2	2	2	2	0	Moderate
[27]	0	0	2	0	0	2	0	Low
[28]	1	0	2	2	1	2	0	Moderate
[29]	1	0	2	2	0	2	0	Moderate
[30]	1	1	2	2	0	2	0	Moderate
[31]	0	0	2	0	2	2	0	Moderate
[32]	0	0	2	2	0	2	0	Moderate
[33]	0	0	2	2	0	2	0	Moderate
[34]	0	0	2	2	2	2	0	Moderate
[35]	0	0	2	2	0	2	0	Moderate
[36]	0	0	2	2	0	2	0	Moderate
[37]	0	0	2	2	0	2	0	Moderate
[38]	0	0	2	0	0	2	0	Low
[39]	0	0	2	2	0	2	0	Moderate
[40]	0	0	2	2	0	2	0	Moderate

### 3.3. Data Analysis

The outcome of this systematic review generated 22 studies. The characteristics of the included papers are presented in Table 3. The inclination toward using a conventional heat cure denture base PMMA polymer was seen as very common in the majority of papers [20,21,22,23,24,25,26,27,28,29,30,31,32,33,34,35,36,37,38,39,40]. However, a few studies focused on experimenting with a chemically modified PMMA polymer by incorporating a monomer either in pre-polymerized polymer powder of PMMA [40] or in unpolymerized MMA liquid [23,29]. CAD-CAM milled resin material was of particular interest to a few investigators [20,24,27]. Inclination towards the use of PEEK material as denture base was seen only in one research paper [33]. Only two papers evaluated the mechanical properties of rubber reinforced PMMA denture base material [19,31].

In the majority of the papers, compression moulding using a water bath was observed as the dominant technique for the polymerization of denture base [19,20,21,22,24,28,29,30,31,32,35,36,37,38,39]. However, the dry heat polymerization method was also explored by some investigators with a promising effect on mechanical properties [27,31]. The use of high pressure during heat polymerization also produced encouraging results on the mechanical properties of denture base material [24,30]. Interestingly, the use of CAD-CAM resin block and milling technique is gaining attention among the investigators [20,24,27] suggesting improved FS [20,24]. In contrast, PEEK as denture base material was used in a single study with improved mechanical properties [33].

Among the other notable polymerization methods used were microwave [21,23,25,26,38,39], injection moulding [20,36,37] autoclave [19,23], air circulating [31,32], and dry heat [31,32].

FS was the most commonly used testing parameter employed to evaluate the mechanical properties [20,23,24,25,26,27,29,30,32,34,35,36,37,39,40]. The investigators were also interested to see TS [19,28,33], SH [19,21,22,24,27,31,38] and IS [19,27,28,31,33,35]. In contrast, FT was evaluated in only one research paper [25].

## 4. Discussion

To our knowledge, this is the first systematic review that attempted to evaluate the mechanical properties using a chemically modified PMMA, newer denture base materials and polymerization methods other than the established and accepted ones.

The denture base is vulnerable to fracture after clinical use, which is a problem and concern in prosthodontics. The two most common causes of denture base fracture are impact failure outside the mouth and flexure fatigue failure inside the mouth. The ability to withstand multidirectional and intricate masticatory loads is a fundamental and essential requirement for a denture base material [41].

The importance of reviewing the mechanical properties of denture base material is justified because of the recent advent of chemically modified PMMA or newer denture base materials and polymerization methods. The mechanical properties of a denture base, however, do not necessarily and entirely indicate its clinical performance. Nevertheless, the clinical life of a denture base is mainly dependent on its mechanical properties.

Regarding the quality of the evaluated papers, we observed a medium to low risk of bias in all the papers. However, the included studies lacked standardization in terms of certain parameters such as the cross-head speed of the device during FS testing, sample dimensions, sample size, sample randomization and blinding of the operator during testing. All these factors make the included papers unique and incomparable due to the lack of standardization. In laboratory papers, the chances of bias are reduced and we may assume the difference in outcome between groups is by chance. Yet the importance of an independent observer cannot be overlooked [42].

This systematic review identified enhanced IS and TS using high impact PMMA denture resin. This type of resin is a copolymer of butadiene-styrene having a good optical property. The rubber particles are well dispersed due to grafting with a methacrylate group, thus covalently bonded into the polymer network [43]. The rubber restricts the crack propagation and improves IS by absorbing a greater amount of energy compared to other reinforced denture base materials [44]. Similarly, higher FS in a study by Abdulwahhab et al. [19] could be attributed to the higher resilience and toughness of the rubber material. The rubber can endure stresses without deformation [45]. Similarly, increased SH values observed by Kiran et al. [31] might suggest the potency of this copolymer. However, further investigation using a copolymer of butadiene-styrene in PMMA resin is necessary to reach any conclusion.

This systematic review identified enhanced FS and FM using CAD/CAM milled resin blocks. The enhanced FS may be attributed to industrial processing techniques capable of providing a homogeneous material with fewer flaws [20,24]. The other investigators have also attributed the higher mechanical properties of CAD/CAM milled resin blocks to reduced voids and flaws than traditional heat polymerized PMMA [46,47]. However, lower SH values might indicate a lower degree of conversion (D_c_) than the traditional polymerization method. In contrast, a 3-D printed denture resin involves the use of a monomer based on acrylic esters that have relatively low double-bond conversion compared with conventional acrylic resins [48].

In recent years, chemical modification through the interconnection of monomers, oligomers, and cross-linking agents with PMMA resin has been improvised, executed and proposed [49,50]. Incorporated oligomers can strengthen the acrylic resin dentures [51] due to the blending effect of two different polymers. By blending two polymers with different physical properties, in different volume ratios, a new material with tunable properties may be formed [8].

In a study by Ayaz and Durkan, higher FS and FM values were observed when acrylamide monomer was prepared with 15% to the molecular weight ratio of the PMMA. The statistically higher FS values suggest that copolymerization of PMMA and acrylamide monomer was successfully formed [23]. Similarly, Consani et al. [25] used hexanediol dimethacrylate (HDDMA) monomer up to 10 wt.% of the MMA liquid as a cross-linking agent. They observed that the addition of HDDMA as a cross-linker provides covalent interactions between the linear PMMA chains. In principle, stabilizing the structure reduces water sorption and solubility and increases flexural properties [25]. However, in Consani et al.’s recent study [26], they found that the oligomeric additive (i.e., thiourethane oligomer) is not suitable, as all mechanical properties were negatively affected. The negative effect might be attributed to the compatibility of the monomers forming the blend, or if the polymer pairs do not react with each other, a material with inferior mechanical properties may be formed [8]. In contrast, the higher FS and FM in the study by Hayran and Keskin suggest that copolymerization of PMMA with ethyl-methacrylate, butyl-methacrylate (BMA), or isobutyl-methacrylate (IBMA) is favourable [29]. According to the investigators, copolymer type and the number of cross-linking agents in the polymer have significant effects on the mechanical properties of denture resins [52].

Tricyclodecane dimethanol diacrylate (TCDDMDA) is a novel cross-linking monomer possessing a tri-ring central group that imparts a steric hindrance effect by slowing down the polymerization rate and facilitating the monomeric conversion to polymer, thus reducing the residual monomer content. It is a dual-reactive monomer and has easily polymerizable carbon-carbon double bonds with a highly reactive pendant acrylate group. The increased FS and IS of the experimental groups can be attributed to the low availability of the pendant acrylate groups after polymerization [35]. Evidence of copolymerization with improved FS was demonstrated without damaging the chemical structure matrix of PMMA, by adding selected compositions of hydroxyethyl methacrylate (HEMA) and IBMA which have similar characteristics to that structure [37]. Similarly, Yang et al. [40] suggested that polyimide macromolecules in low loading (i.e., 0.6 wt%) could show significantly high FS by 13.5% compared to the control group.

Polyetheretherketone (PEEK) is a semi-crystalline thermoplastic material that could be considered an innovative material to replace PMMA [33]. Both PEEK specimens milled and pressed at 200 °C mould temperature had higher tensile strength. PEEK polymer could be considered as a resistant material to notch concentration as it revealed higher Izod impact strength than the PMMA. However, the findings cannot be accepted based on a single study. Similarly, the use of ceromers as inorganic-organic hybrid polymeric materials has been advocated. These materials are used as coating agents for scratch resistance and can significantly increase the FS and FM of the PMMA denture resin. The functional group of the ceromer structure binds to the polymer structure, and the other part contributes to enhancing the hardness and wear resistance to the surface [28,53]. However, further research is necessary to ascertain any kind of causal relationship.

We observed that compression moulding using a water bath is still being employed as a widely used denture base fabrication technique (REFs). However, a recent inclination toward using microwave energy suggests that this method has the potential to demonstrate similar results to conventional denture processing [26], with the advantage of faster processing times [25]. The principle of the use of microwave energy depends on the effect of microwave energy on the monomer components promoting a uniform and immediate heating of the polymer mass, that activates the decomposition of benzoyl peroxide, and quickly yields free radicals for the polymerization process, which decreases in the same proportion as polymerization increases [21]. However, time/power combination is important and it should be 650 W for 5 min for optimal results [38]. The findings of Consani et al. [25,26] must be interpreted with caution as no other polymerization method was used in their studies to compare. In contrast, Ayaz and Durkan found microwave energy as successful as the autoclave polymerization method [23]. While comparing and contrasting microwave energy with compression moulding using a water bath, Kumar et al. advocated improved IS using the microwave energy method [21]. Through microwave polymerization, flexural properties such as that of a compression moulding water bath technique can be achieved [39].

Autoclaving seems to be another polymerization technique through which improved mechanical properties of denture base polymer can be achieved. The autoclave polymerization technique increases the D_c_ and decreases residual monomers [54]. However, slow (long) curing is essential for complete polymerization and improved mechanical properties [19]. While evaluating the effectiveness of the autoclaving polymerization technique on three different commercially available denture base resins, Ayaz et al. deduced that even the fast curing method significantly reduces the number of residual monomers and increases the hardness of the polymerized denture resin compared to conventional compression moulding water bath technique [22].

Injection moulding is a new technique that allows a controlled polymerization process due to the flask design. A continuous flow of material from the sprue compensates for polymerization shrinkage and results in a dimensionally accurate denture than that produced by compression moulding [55]. A higher FS and homogenous resin surface without voids at the microscopic level can be achieved with the injection moulding technique [36]. In contrast, lower FS values were observed when the effectiveness of injection moulding was compared and contrasted with the conventional compression moulding technique [37]. Similarly, reduced FS and FM in the injection moulding group compared to compression moulding suggest that this type of polymerization technique is not ideal for denture base polymerization [20]. Further studies are required to reach any conclusion.

The beginning of a new century has witnessed the use of CAD/CAM for dentures fabrication [56]. The CAD/CAM materials are not only esthetically pleasing but also durable [57]. Moreover, their processing is efficient, fabrication is quick and marginal fit is accurate [58]. The mechanical durability and clinical life of such prostheses are predictable [59]. The higher FS values can be attributed to CAD-CAM resins that are prepolymerized discs polymerized by using sophisticated equipment capable of providing greater polymerization potential [20], with fewer flaws [24]. However, reduced SH values might indicate lower Dc of the discs [24]. While poor mechanical properties using a 3D technique might be attributed to layering built in a direction parallel to the load direction and weak adhesion between successive layers results [27].

High-pressure polymerization is a new technique that positively influences the conversion of the monomers into high-molecular-weight polymers by increasing the Dc [60]. In free-radical polymerization, high pressures greatly increase Dc, with an enhanced propagation rate constant and reduced termination rate constant [30]. High pressure up to 500 MPa can increase the FT due to the increased polymerization rate resulting in an increase in the molecular weight of the polymer [34]. However, if the pressure is increased further, the monomers are likely to transform into solids and form monomer crystals, thus reducing the D_c_ and hence reducing flexural properties [34].

Kiran et al. advocated the benefits of the air-circulating oven for its homogenous distribution of hot air circulating chamber and their improved IS and SH properties might support the idea of using this new device for denture polymerization [32]. However, in another study, reduced FS compared to the water bath polymerization technique creates doubt about this new system [32].

Based on the findings of this comprehensive study, we assume that if low molecular weight chemical modifiers are used in optimal concentration, the mechanical properties of PMMA based denture material can be significantly improved due to a decrease in polymeric shrinkage and stress [26,61]. The copolymerization of PMMA with low molecular weight monomers increases the mechanical properties by increasing the cross-linking in the polymer network [29]. We assume that appropriate chemical modifiers can drastically improve the compromised mechanical properties of the PMMA denture base. However, further research in this area is necessary. In contrast, limited studies on the effectiveness of the contemporary polymerization methods do not warrant their superiority over the traditional compression moulding water bath technique. Though CAD/CAM technique might have the potential to overtake the traditional polymerization method, this method is still in its infancy.

This systematic review mainly focused on experimental denture polymers and unconventional polymerization methods for enhanced denture base mechanical properties. Other review papers were available on the subject matter at the time of this systematic review [20,49,62,63,64], however, either the papers reviewed the effect of incorporated fillers or chemical modification of a PMMA denture resin or the review was limited to one or two unconventional polymerization methods. In contrast, this paper systematically reviewed and thoroughly discussed the experimental denture materials (either chemically modified PMMA or newer materials) that have been used recently and the unconventional denture fabrication techniques. For explicitness and comprehensibility, every selected study was tabulated describing the testing method, denture base material used, polymerization method employed, and the outcomes achieved.

The results of this systematic review necessitate cautious interpretation since laboratory experiments have inherent limitations while clinically the material functions differently due to oral conditions such as masticatory loads, masticatory cycles, temperature fluctuations, microbial flora, and salivary flow rate. Clinical trials with long follow-up periods are necessary to infer a conclusion.

## 5. Conclusions

It is difficult to draw any conclusion concerning the effectiveness of newer denture base materials and polymerization methods. However, despite the limitations of this research work, the findings provide evidence that the chemical modification of PMMA resin through the interconnection of monomers, oligomers, and cross-linkers provides covalent interactions and stabilizes the PMMA structure. Hence, reduced water sorption, solubility and increases flexural properties are witnessed. CAD/CAM milled resin blocks and CAD/CAM technique might be a useful alternative in enhancing the mechanical properties of the denture base but their clinical use needs further trials and investigations. Despite so many available polymerization methods and techniques, compression moulding using a water bath produces an acceptable mechanical outcome. The other innovative methods are still in the initial trial stages and hence need further laboratory evaluation.

## Figures and Tables

**Table 1:** Search plan/approach.

Source	Criteria
Database	Medline/Pubmed, Web of Science, Scopus
Date of publication	01 January 2011–31 December 2021
Keywords	Experimental denture polymer Novel denture polymer Reinforcement of denture base
language	English
Type of paper	in vitro study/analysis
Inclusion criteria	Mechanical properties of newer denture base material or polymerization method
Exclusion criteria	Studies related to meta-analysis, review, case report/series, biological/chemical/physical and thermal, clinical trial, denture repair/lining, overdenture, denture teeth, implant/finite element analysis/fixed prosthesis, and filler/fibre reinforced denture base
Journal category	Dental, Medline, Materials science

**Table 3:** Included papers with the type of denture base polymer, polymerization method and their corresponding outcomes.

Reference	Testing Method	Denture Base Material	Polymerization Method	Outcome
[19]	SH, FS, IS	High impact PMMA	Compression moulding using a water bath, autoclave	↑↑ SH, FS and IS in water bath processing and slow autoclave processing groups
[20]	FS, FM	Heat cure PMMA, CAD-CAM milled resin	Compression moulding using a water bath, injection moulding, CAD-CAM milling	↑↑ FS, FM of the CAD-CAM milled groups
[21]	IS	Heat cure PMMA	Compression moulding using a water bath, microwave	↑↑ in IS of microwave technique compared to water bath group
[22]	SH	Heat cure PMMA	Compression moulding using a water bath, autoclave for 10 min, autoclave for 20 min	↑↑ SH in both 10 min and 20 min autoclave polymerization groups
[23]	FS, FM	Heat cure PMMA, 5%, 10%, 15% and 20% acrylamide monomer in heat cure PMMA	Autoclave, microwave	↑↑ FS in 15% copolymer group
[24]	FS, SH	Heat cure PMMA, CAD-CAM block	Compression moulding using a water bath, heat polymerization at 100 °C under high pressure (200 MPa), CAD-CAM milling	↑↑ FS while ↓↓ SH in CAD blocks.
[25]	FS, FM, FT	Heat cure PMMA with and without Hexanediol dimethacrylate HDDMA (10, 20, 30 wt%) and TU (10 wt%)	Microwave	10 wt% HDDMA ↑↑ the mechanical properties (FS, FM & FT) of denture base resin
[26]	FS	Heat cure PMMA with or without TU in various wt%	Microwave	FS ↓ as glass filler uploading ↑
[27]	FS, IS, SH	Heat cure PMMA, 3-D printed denture resin	Compression moulding using a water bath, 3-D printing	↑↑ FS, IS & SH in Compression moulding groups
[28]	TS, EM, IS	Heat cure PMMA, heat cure PMMA coated with ceromers	Compression moulding using a water bath	Coating with ceromers ↑↑ the mechanical properties of PMMA denture base
[29]	FS, FM	Heat cure PMMA, heat cure PMMA copolymerized with EMA, BMA, and IBMA	Compression moulding using a water bath	FS & FM values of all copolymer groups were ↑ than those of the control group
[30]	FS	Heat cure PMMA	High-pressure dry curing, compression moulding using a water bath	↑↑ FS in samples fabricated in a dry environment at high pressure
[31]	SH, IS	Heat cure PMMA, high impact PMMA	Compression moulding using air circulating oven, dry heat, water bath	↑ SH & IS in rubber reinforced PMMA using air circulating oven and dry heat oven
[32]	FS	Heat cure PMMA	Compression moulding using air circulating oven, dry heat, water bath	↑↑ FS in water bath group
[33]	IS, TS, EM	Heat cure PMMA, PMMA-pressed, PEEK	PEEK-pressed (100 °C, 150 °C, 175 °C & 200 °C) & PEEK-milled	↑↑ TS & EM in PEEK-milled groups. While ↑ IS in PEEK-pressed at 100 °C
[34]	FT, FS, EM	Heat cure PMMA	High pressure polymerization at 500, 800 & 980 MPa	↑↑ FT and ↓↓ FS & EM in high pressure polymerized groups compared to ambient temperature polymerized control group
[35]	FS, IS	Heat cure PMMA, heat cure PMMA with tricyclodecane dimethanol diacrylate comonomer at 10% and 20% (*v/v*)	Compression moulding using a water bath	↑↑ FS & IS in experimental groups
[36]	FS	Heat cure PMMA	Compression moulding using a water bath, injection moulding thermo-pressed	↑↑ FS in injection moulded a thermo-pressed group
[37]	FS	Heat cure PMMA with and without IBMA and HEMA monomers	Compression moulding using a water bath, injection moulding thermo-pressed	Low wt.% of IBMA or HEMA ↑↑ FS
[38]	SH	Heat cure PMMA	Compression moulding using a water bath, microwave (at 550 W, 630 W or 650 W)	↔ in SH of the control and experimental groups
[39]	FS, FM	Heat cure PMMA	Compression moulding using a water bath, microwave (at 550 W, 630 W, 650 W or 700 W)	↔ in FS & FM of the control and experimental groups
[40]	FS	Heat cure PMMA, heat cure PMMA with 0.4, 0.6, 0.8 and 1 wt% polyimide monomer	Compression moulding with heat polymerization at 100 °C for 1 h	↑↑ FS and FM using low wt.% of polyimide monomer in PMMA

Key: ↑↑ = significant increase, ↑= increase, ↔ = no significant change, ↓ = decrease, ↓↓ = significant decrease, FS = flexural strength, SH = surface hardness, IS = impact strength, FT = fracture toughness, FM = flexural modulus, TS = tensile strength, EM = elastic modulus, PMMA = polymethyl methacrylate, CAD-CAM = computer aided design-computer aided manufacturing, CAD = computer aided design, HDDMA = hexanediol dimethacrylate, TU = Thiourethane, EMA = ethyl methacrylate, BMA = butyl methacrylate, IBMA = isobutyl methacrylate, HEMA = hydroxyethyl-methacrylate.

## Data Availability

No data.
